# The risk factors associated with pulmonary hypertension in patients with left heart diseases

**DOI:** 10.1097/MD.0000000000047799

**Published:** 2026-02-20

**Authors:** Kaina Wang, Jin Fan, Ling Mei

**Affiliations:** a Department of Endocrinology, Shanghai Putuo District Liqun Hospital, Shanghai, China; b Department of Geriatric Caridovascular, Chinese People’s Liberation Army (PLA) General Hospital, Beijing, China.

**Keywords:** elderly patients, left heart disease, pulmonary hypertension, risk factors

## Abstract

Pulmonary hypertension (PH) is an important reason for morbidity and mortality in patients with left heart disease (LHD). LHD is one the most common cause of PH in the elderly. Few studies have reported risk factors in patients with PH accompanied with LHD. To identify associated risk factors with PH in patients with LHD in elderly. A total of 411 aged male patients (>60 years) with LHDs were enrolled in this trail. Pulmonary artery systolic pressure (PASP), heart chamber diameters, and left ventricular functions were evaluated by transthoracic echocardiography. Patients were classified as PH group (n = 211) and non-PH group (n = 200) according to their PASP. Clinical history was collected from medical recordings. Independent associated factors for PH were identified by Logistic regression analysis. Compared with non-PHs, PH patients were more likely to have atrial fibrillation (AF; 21% vs 9.4%, *P* < .01), left atrial enlargement (39.25 ± 4.96 mm vs 36.94 ± 3.16 mm, *P* < .01), and reduced left ventricular ejection fraction (LVEF; 58.97 ± 6.51% vs 61.15 ± 4.72%, *P* < .01). Multivariate logistic regression analysis showed that AF, larger left atrial diameter, lower LVEF was independently associated with the presence of PH in patients with LHDs. In patients with PH, the PASP and left atrial diameter were all related to the number of co-morbidities (*P* < .05). AF, left atrial enlargement, and decreased LVEF are associated with the presence of PH in patients with LHDs. Well control of comorbidities and underlying heart diseases, prevention or treatment of AF may play key roles in the management of PH in patients with LHDs.

## 1. Introduction

Pulmonary hypertension (PH) is the end result of a variety of patients with heart diseases during diverse pathologic processes. The chronic elevation of pulmonary artery pressure often leads to right ventricular pressure overload and subsequent the right ventricular failure. PH due to left heart disease (LHD) is classified as group II according to the Dana Point classification,^[1]^ which includes left ventricular (LV) systolic and/or diastolic left heart failure, and left-sided valvular disease.

LHD is the most frequent cause of PH. It has been reported that there were more than 60% of patients with LV systolic dysfunction and 80% of patients with LV diastolic dysfunction who had the PH.^[2]^ In patients with left-sided cardiac disease, the presence of PH is associated with increased morbidity and mortality.^[3,4]^ the presence of PH was significantly associated with increased in-hospital mortality in HFpEF patients.^[5]^ It also has long been observed by community-based studies that the existence of secondary PH is associated with worse prognosis in HF.^[3,6]^ Some reports have showed that pulmonary artery systolic pressure (PASP) increases more likely in accordance with age.^[7,8]^ As the population ages rapidly, the number of patients presenting with PH is also increasing.

In this study, the characteristics, influence and prognostic factors of patients with PH and LHD were analysis. The significance of PH in elderly male patients was also investigated. This study aims to determine the clinical risk factors in patients with PH-LHD. Considering the number of recent advances in the management of PH, it is likely that a better understanding of the impact of risk factors on PH-LHD might assist the clinical management of patients with PH.

## 2. Methods

This study conformed to the ethical guidelines of the Declaration of Helsinki as reflected in a priori approval by the human research committee of Shanghai Putuo District Liqun Hospital. The written informed consent was acquired from each patient.

### 2.1. Study population

Patients with elder than 60 (including 60) were enrolled in this study from December 2007 to December 2010 in China hospital. The control group was the patients without PH by heart catheterization or echocardiography. Both of the groups have the same demographic characteristics.

The inclusion criteria were as follows,

1. 60 years or older;
2. LHD;
3. Echocardiogram: Left ventricular ejection fraction (LVEF) ≤ 40%, or E/A<0.8.

The exclusion criteria were as follows,

1. History of idiopathic pulmonary arterial hypertension;
2. Chronic obstructive pulmonary disease;
3. Right heart failure;
4. Liver or renal failure;
5. Malignancy.

The complications were defined as follows:

1. Hypertension: blood pressure ≥ 140/90 mm Hg or a history of hypertension and being on antihypertensive medications;
2. Diabetes mellitus: fasting blood sugar > 125 mg/dL or being on antidiabetic agents;
3. Coronary artery disease (CAD): presence of a history angina pectoris, myocardial infarction, a positive stress test, angiographic evidence CAD, coronary intervention, coronary artery bypass surgery or presence of significant Q-waves on the surface electrocardiogram.
4. Hyperlipidemia: defined as plasma triglycerides 150 mg/dL or high-density lipoprotein cholesterol 40 mg/dL in men or 50 mg/dL in women and patients who taking cholesterol lowering medications.

### 2.2. Echocardiography

All echocardiograms were obtained by an experienced sonographer by using cardiac ultrasound (SIEMENS Sequoia512, Siemens Medical Solutions USA, Inc., Mountain View). Images were stored digitally using commercial software (Prosolv Cardiovascular, Indianapolis) and reported by a senior echocardiologist in according with published guidelines. Standard complete 2-dimensional echocardiography was performed in all individuals, with chamber quantification assessed according to published criteria. As PASP is equal to right ventricular systolic pressure in the absence of pulmonary stenosis, PASP was assessed by imaging the peak velocity of the tricuspid regurgitant jet and applying the simplified Bernoulli equation (PASP = 4v^2^ + right atrial pressure). PH was defined as PASP ≥ 40 mm Hg.

For standardization, a right atrial pressure of 10 mm Hg was assumed for all patients unless clear features were present that suggested otherwise. Insufficient tricuspid regurgitation was defined as incomplete tricuspid regurgitant jet velocity or inability to identify peak velocity with confidence.

### 2.3. Statistics

Quantitative variables are described using mean (± standard deviation). Cross tabulations using the *x*^2^ statistic were used with nonparametric Kruskal–Wallis test to compare differences. Independent clinical and echocardiographic determinants of PH were assessed using logistic regression with backward elimination of independent variables based on likelihood-ratio tests. Analyses were performed using SPSS17.0 (SPSS Inc, Chicago). *P*-values were considered significant if < .05.

## 3. Results

### 3.1. Demographic characteristics of patients with and without PH

Comparisons of patients with and without PH are shown in Table 1. There are not significantly difference between 2 groups smoking, drinking, have the history of coronary heart disease, hypertension, diabetes, and hyperlipidemia). But patients with PH often have the history of atrial fibrillation (AF; 21% vs 9.4%, *P* < .01).

**Table 1:** Baseline characteristics of patients between 2 groups ($\bar{x}\pm \;s$).

	PH (n = 200)	NPH (n = 211)	*P*
Age (yrs)	82.67 ± 7.62	81.30 ± 7.22	.620
Body mass index (kg/m^2^)	24.48 ± 2.71	24.84 ± 2.75	.197
Smoking history, n (%)	56 (28.0)	58 (27.5)	.838
Drinking history, n (%)	18 (9.0)	22 (10.4)	.276
Medical history			
Coronary artery disease, n (%)	153 (76.5)	151 (71.6)	.254
Hypertension, n (%)	165 (82.5)	186 (88.2)	.105
Diabetes mellitus, n (%)	73 (36.5)	74 (35.1)	.763
Hyperlipidemia, n (%)	37 (18.5)	43 (20.4)	.631
Atrial fibrillation, n (%)	42 (21.0)	20 (9.4)	.001*
RHD n (%)	2 (1.0)	0	
DCM, n (%)	1 (0.5)	0	
HCM, n (%)	1 (0.5)	0	

DCM = dilated cardiomyopathy, HCM = hypertrophic cardiomyopathy, NPH = non-pulmonary hypertension, PH = pulmonary hypertension, RHD = rheumatic heart disease.

* *P* < .05, significantly difference.

### 3.2. Echocardiographic variables

Echocardiographic variables are shown in Table 2. In patients with PH, LA area, RV area, and RA area were all markedly increased, LVEF and main pulmonary artery (MPA) decreased. Right pulmonary artery (RPA), LV end diastolic dimension, LV end systolic dimension, LV fraction shortening, LV posterior wall distance, and interventricular septum had no significantly difference compared with control group.

**Table 2:** Echocardiographic variables in patients with PH and without PH ($\bar{x}\pm \;s$).

	PH (n = 200)	NPH (n = 211)	*P*
MPA (mm)	24.30 ± 2.20	24.82 ± 1.77	.012*
RPA (mm)	14.85 ± 1.64	15.11 ± 1.57	.127
LAD (mm)	39.25 ± 4.96	36.94 ± 3.16	.000*
LVEDD (mm)	48.81 ± 4.41	48.82 ± 3.78	.981
LVESD (mm)	33.12 ± 4.19	32.60 ± 3.12	.184
RVD (mm)	36.08 ± 2.61	35.09 ± 2.05	.000*
RAD (mm)	38.59 ± 3.89	37.15 ± 2.27	.000*
LVFS (%)	32.55 ± 2.77	33.38 ± 2.66	.005*
LVEF (%)	58.97 ± 6.51	61.15 ± 4.72	.000*
LVPWT (mm)	10.29 ± 1.17	10.32 ± 1.14	.792
IVST (mm)	10.90 ± 1.61	10.80 ± 1.26	.155

IVST = interventricular septum, LAD = left atrial dimension, LVEDD = left ventricular end diastolic dimension, LVEF = left ventricular ejection fraction, LVESD = left ventricular end systolic dimension, LVFS = left ventricular fraction shortening, LVPWT = left ventricular posterior wall distance, MPA = main pulmonary artery, NPH = non-pulmonary hypertension, PH = pulmonary hypertension, RAD = right atrial dimension, RPA = right pulmonary artery, RVD = right ventricular dimension.

* *P* < .05, significantly difference.

### 3.3. Effect of age on PASP and LVEF in patients with PH

The 200 patients with PH were divided into 3 groups: 16 between 60 and 69 years (Group A), 42 between 70 and 79 years (Group B),142 older than 80 years (Group C). There was a significantly decrease in LVEF according with age. However, older patients had higher pulmonary artery pressure. While PASP, MPA, RPA, left atrial dimension (LAD), LV end diastolic dimension, LV end systolic dimension, right atrial dimension, right ventricular dimension didn’t show significantly among these groups (Table 3).

**Table 3:** Effect of age on PASP and LVEF in patients with PH ($\bar{x}\pm \;s$).

	Group A (n = 16) ①	Group B (n = 42) ②	Group C (n = 142) ③	*F*	Multiple comparisons
LVEF (%)	61.88 ± 6.21	61.40 ± 6.355	57.89 ± 6.33	6.883**	③<②
PASP (mm Hg)	43.94 ± 5.67	46.74 ± 8.710	46.80 ± 7.15	1.090	

LVEF = left ventricular ejection fraction, PASP = pulmonary hypertension systolic pressure, PH = pulmonary hypertension.

* Indicates a statistically significant difference, *P* < .05.

** *P* < .01, significantly difference.

### 3.4. The analysis of echocardiographic in patients with PH according with complications cohort

The patients with PH were divided into 4 groups according with complications, which were coronary heart disease, hypertension, diabetes mellitus, hyperlipidemia, and AF. These groups were 1, 2, 3, and 4 complications disease, which means patients have 1, 2, 3, and 4 complications disease. The characteristics of echocardiographic were analyzed. The results showed that, the pulmonary arterial systolic pressure (*F* = 2.898, *P* < .05) and diameter of left atrial (*F* = 5.063, *P* < .05) were significantly arisen according with the numbers of the complications in degree. The pulmonary arterial systolic pressure in group of 4 complications was significantly higher than single complications groups. The diameter of left atrial in 3 and 4 complications group were significantly higher than single complications group (*P* < .05). And the diameter of left atrial in 4 complications group was significantly higher than 2 complications group (*P* < .05). The inner diameter of MPA, RPA, right atrium, right ventricle, left ventricle, and ejection fraction of left ventricle had no significantly difference in each group (Table 4).

**Table 4:** The subgroup analysis of characteristics of cardiac ultrasound in PH patients with the impact of complication disease ($\bar{x}\pm \;s$).

	Single disease group (n = 35) ①	Double disease group (n = 72) ②	Three disease group (n = 71) ③	Four disease group (n = 22) ④	*F*	Multiple comparisons
LAD (mm)	37.46 ± 3.60	38.63 ± 4.51	39.87 ± 5.28	42.14 ± 5.78	5.063*	④③>①; ④>②
LVEF (%)	59.57 ± 6.72	59.38 ± 6.37	59.01 ± 6.25	56.50 ± 7.33	1.253	
PASP (mm Hg)	44.34 ± 4.63	46.71 ± 9.10	46.38 ± 6.06	50.18 ± 7.85	2.898*	④>①

The cox correlation of PH patients with LHD.

LAD = left atrial dimension, LHD = left heart disease, LVEF = left ventricular ejection fraction, PASP = pulmonary hypertension systolic pressure, PH = pulmonary hypertension.

* *P* < .05.

** Indicates a statistically significant difference, *P* < .01.

The independent predictors of PH among the 411 patients with LHD were: larger left atrial diameter, lower LVEF, and the history of AF. Age, smoking, hypertension, diabetes, CAD, body mass index, and hyperlipidemia were not independent predictors (Table 5).

**Table 5:** Logistic regression studying the determinants of PH in patients with LHD.

Clinical and echocardiographic variables	OR	SE	*P*	95% CI
Atrial fibrillation	2.782	0.334	.002*	1.447–5.348
LAD (mm)	2.753	0.233	.000*	1.742–4.350
LVEF (%)	3.436	0.538	.022*	1.196–9.871

LAD = left atrial dimension, LHD = left heart disease, LVEF = left ventricular ejection fraction, PH = pulmonary hypertension.

* *P* < .05, significantly difference.

## 4. Discussion

The characteristics and impact factors of LHD patients with and without PH were analyzed in this study. We found that smoking, drinking, have the history of CAD, hypertension, diabetes, and hyperlipidemia had no significantly difference in LHD patients with or without PH. Patients with PH were more likely had larger LAD and the history of AF.

AF is the most common sustained cardiac arrhythmia among elderly people. The hemodynamic factors that might contribute to onset of AF in PH were as follows. Previously, AF reduced LV function, and elevated end-diastolic LV pressure, which in turn leads to higher left atrial pressures and larger left atrial diameter.^[9,10]^ Increased left atrial pressure might affects atrial systolic function, above all, atrial compliance, and hence can lead to higher pulmonary venous pressures.

In our study, we found that patients with PH had the history of AF more than patients who without PH, supporting the notion that the prevalence of AF in PH is considerably higher than in the normal population at similar age.^[11,12]^ We found AF was a powerful risk factors of PH, this was also true for all PH of various origins,^[13]^ indicating that in PH related to LHD AF might connections to or further development of PH. In spite of there are no evidence of the PH-LHD patients can profit from ablation of medically refractory arrhythmias, some studies have shown that congenital heart disease patients with PAH may benefit from it. These findings might have clinical implications. It is requiring confirmation by a larger, multicenter evaluation study in future.

Previous studies have reported that age was the strongest predictor of PASP.^[14]^ showed that there are strong correlations between PASP and age (*r* = 0.35, *P* = .011).^[15,16]^ As in prior studies, we found LAD is substantially larger with advancing age and PASP increased in cohort analysis, these changes in PASP with advancing age are consistent with the hypothesis that with the age advanced, PASP may affected by arterial stiffness, LV diastolic dysfunction, and decreased left atrial compliance in geriatric age male patients who with PH accompanied with LHD.

In clinical practice, the association between multiple factors and PH is a significant problem. However, there is little known about it. Kogan et al examined the prevalence of obesity and metabolic syndrome in patients with severe PH.^[17]^ They found that 11% of patients were morbidly obese without significant cardiac, pulmonary or vascular causes of PH. This group was characterized by high incidence of diabetes mellitus, arterial hypertension, hyperlipidemia, AF and LV diastolic dysfunction. Robbins IM found that 70.6% of PVH patients had 3 or more features of the MS vs 20.0% of PAH patients (*P* = .001; OR: 9.6; 95% CI: 2.5–36.4).^[18]^ The patients in our study were with more comorbidities may have higher grades of PASP, larger LAD. It is suggested that multiple factors may impacted pulmonary circulation and left heart diastolic function, which in turn leads to aggravate PH. There is a high prevalence of metabolic syndrome in elder patients.^[19]^ Metabolic syndrome has become a noteworthy public health problem. Thus, we suggest that the integrated control of hypertension, diabetes and AF should be priority in heart disease patients with PH, especially for male older patients.

## 5. Conclusion

The degree of PASP increased may be correlated with the number of complications in elderly male patients. AF, left atrial enlargement, and decreased LVEF are associated with the presence of PH in patients with LHDs. Well control of comorbidities and underlying heart diseases, prevention or treatment of AF may play key roles in the management of PH in patients with LHDs.

## 6. Limitation

First, although transthoracic echocardiography serves as the cornerstone noninvasive modality for estimating systolic pulmonary artery pressure and screening for PH, it is subject to measurement inaccuracies. Prior studies have reported that transthoracic echocardiography may lead to either overestimation^[20]^ or underestimation^[21]^ of systolic pulmonary artery pressure due to technical limitations and patient-specific factors. Second, this was a single-center, cross-sectional study with a relatively small sample size, which may limit the generalizability of our findings. To further validate the potential of AF, left atrial enlargement, and reduced LVEF as early warning indicators, future large-scale, multicenter longitudinal studies with adequate follow-up in middle-aged and elderly populations are warranted to ensure robust statistical significance.

## Author contributions

**Conceptualization:** Kaina Wang, Jin Fan.

**Data curation:** Jin Fan.

**Formal analysis:** Jin Fan.

**Funding acquisition:** Kaina Wang, Jin Fan.

**Investigation:** Kaina Wang.

**Methodology:** Jin Fan.

**Software:** Jin Fan.

**Supervision:** Kaina Wang.

**Writing – original draft:** Kaina Wang.

**Writing – review & editing:** Kaina Wang, Ling Mei.
